# Finding Risk Groups by Optimizing Artificial Neural Networks on the Area under the Survival Curve Using Genetic Algorithms

**DOI:** 10.1371/journal.pone.0137597

**Published:** 2015-09-09

**Authors:** Jonas Kalderstam, Patrik Edén, Mattias Ohlsson

**Affiliations:** Department of Astronomy and Theoretical Physics, Lund University, Lund, Sweden; Jiangnan University, CHINA

## Abstract

We investigate a new method to place patients into risk groups in censored survival data. Properties such as median survival time, and end survival rate, are implicitly improved by optimizing the area under the survival curve. Artificial neural networks (ANN) are trained to either maximize or minimize this area using a genetic algorithm, and combined into an ensemble to predict one of low, intermediate, or high risk groups. Estimated patient risk can influence treatment choices, and is important for study stratification. A common approach is to sort the patients according to a prognostic index and then group them along the quartile limits. The Cox proportional hazards model (Cox) is one example of this approach. Another method of doing risk grouping is recursive partitioning (Rpart), which constructs a decision tree where each branch point maximizes the statistical separation between the groups. ANN, Cox, and Rpart are compared on five publicly available data sets with varying properties. Cross-validation, as well as separate test sets, are used to validate the models. Results on the test sets show comparable performance, except for the smallest data set where Rpart’s predicted risk groups turn out to be inverted, an example of crossing survival curves. Cross-validation shows that all three models exhibit crossing of some survival curves on this small data set but that the ANN model manages the best separation of groups in terms of median survival time before such crossings. The conclusion is that optimizing the area under the survival curve is a viable approach to identify risk groups. Training ANNs to optimize this area combines two key strengths from both prognostic indices and Rpart. First, a desired minimum group size can be specified, as for a prognostic index. Second, the ability to utilize non-linear effects among the covariates, which Rpart is also able to do.

## Introduction

Estimating patient specific risk is often a goal in survival analysis. Common approaches include nomograms and prognostic indices. The models behind these are typically linear in nature—for example using the well known Cox proportional hazards [1] (Cox) model. Various machine learning approaches are also common in this space. Van Belle et al. [2] used support vector machines to calculate a prognostic index. A prognostic index has also been optimized using artificial neural networks [3]. In all these cases each patient is assigned a predicted index, from which the risk is estimated.

Typically, the clinician is not interested in comparing the predicted risk for each patient on an individual basis. Instead, the more general question “does the patient have *high* or *low* risk” is of interest as it relates to the treatment decision, where the clinician selects among a limited number of treatment strategies. Using a prognostic index, a common approach is to choose one or two cuts according to the quartiles. The lower quartile is deemed low risk, the upper quartile high risk, and the rest to be an intermediate group. An approach to automatically determine such cuts, given a prognostic index, has been developed by Van Belle et al. [4].

Another approach compared to defining cuts on prognostic indices is to generate the risk groups directly. For this, a well known method is regression trees adapted for censored data [5], also known as recursive partitioning (Rpart), which attempts to find statistically different groupings in the data. All parameter values are explored in order to find the split which generates the most statistically significantly different groups, and then the process is recursively applied until some minimum group size is achieved.

Intuitively, directly predicting the risk group of a patient should be an easier optimization task than first predicting an individual prognostic index and then defining cuts to produce a grouping. In the case of a prognostic index that optimizes the concordance index [6], the order of essentially all patients is important. In a final split into high and low-risk groups however, the order of the patients within the groups is not important. Non-linear effects in the data could be easier to explore when predicting risk groups directly.

To optimize on risk grouping we must define what constitutes a good grouping. If there is no censoring present in the data, then the ideal grouping can be defined directly by sorting the patients according to survival time and labeling the patients to create desired group sizes. It would then be possible to train any classifier on these labels. Most data sets in survival analysis is however censored, and often quite significantly (28% to 86% in the data sets used in this article). It is not possible to know which label to assign to the censored cases, which makes ordinary classifiers difficult to use for this problem. With survival data, where censoring prevents a pre-defined labeling, the performance of a classifier can be judged based on the survival curves (e.g. Kaplan-Meier plots) of its predicted groups. This strategy is used by Rpart [5] by maximizing the separation between groups.

Artificial neural networks (ANN) has been gaining interest in the medical community for quite some time now, and has proven useful for many clinical decision problems [7, 8], including cancer diseases [9, 10]. The rather recent developments in deep learning techniques for ANN [11] have further boosted this machine learning tool, especially in the area of big data. In this study we use ANNs as classification models, where the approach is to train ANNs to identify low-risk (high-risk) groups by maximizing (minimizing) the area under the survival curve. Optimizing the area will implicitly optimize properties such as median survival time and end survival rate, which are typically used to compare the risk groups predicted by different models. An advantage of ANN models, compared to other machine learning tools such as support vector machines or fuzzy systems, is the use of genetic algorithms in the training process. Optimizing directly on the area under the survival curve cannot be achieved using standard gradient decent based methods. Furthermore, multiple ANNs are combined into an ensemble capable of predicting high, low, or intermediate risk groups. To validate our approach, we compare ANN with Rpart and Cox on five publicly available data sets with different sizes and properties.

## Methods and Materials

### Comparing risk groups

Since correct labels cannot be assigned to censored cases, normal classification metrics such as accuracy, sensitivity, and specificity cannot be used compare the performance of the models. Instead, as is the norm in survival analysis, the survival curves of the predicted risk groups are compared. Some ambiguities regarding what constitutes a “better” risk grouping still have to be resolved:

- If two low-risk groupings (or high-risk groupings) have identical survival curves, we consider the largest grouping to be better. This is akin to both models having the same precision, but different recall.
- If two groupings (both being either high or low risk) have the same size, the best grouping is evident from the survival curves. A better low-risk group would have higher median survival time and/or higher end survival rate for instance.

**Fig 1:**
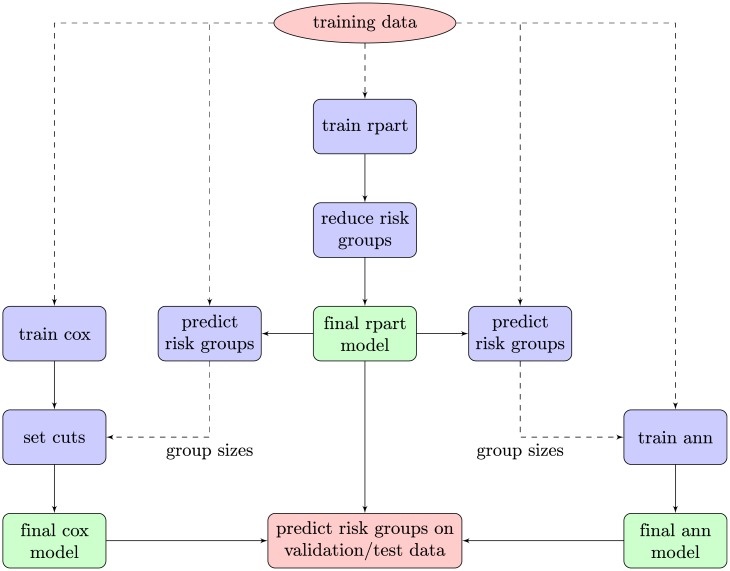
Schematic of the training procedure. It is important to have similar group sizes so that the properties of the survival curves can be compared. To be able to include Rpart in the comparison, it is necessary to compensate for its inability to pre-determine suitable group sizes. The predicted group sizes on the training data (only sizes of groups, not the actual predictions) are set as parameters on Cox and ANN to generate similarly sized risk groups later on the test/validation data.

It is important that the predicted risk group sizes are similar in size to avoid the situation of both different survival curves and different group sizes. Only then is it possible to compare the properties of each risk group between the models. This poses a challenge since ANN and Cox are flexible in terms of group sizes (it can be regarded as a configuration parameter), while the group sizes predicted by Rpart cannot be configured ahead of time. To still be able to include Rpart in the comparison, ANN and Cox are configured with the group sizes which Rpart generates during the training phase, see Fig 1. This ensures that all of the models generate predicted risk groups on the test/validation sets of similar size. In other circumstances, the group sizes configured on Cox and ANN would be up to the clinician, but quartile limits seem to be common in the literature.

### Rpart and Cox

Both Rpart and Cox models were trained and evaluated using the R statistical environment [12]. Rpart generally produces more than three groups, which is the focus in this paper. Thus, to reduce the number of groups, we iteratively joined groups that were not significantly different by the log-rank test starting from the groups with the highest/the lowest survival, along the lines of Banerjee et al. [13]. In case these groups were too small, we manually combined adjacent groups until high and low-risk groups approached one quartile in size. All the remaining groups were labeled as intermediate risk. Using this procedure, small deviations from the requirement of one quartile high and low-risk groups had to be accepted. Experiments with different parameter values for Rpart were performed, but the default parameters [14] worked the best and were hence used for all tests in this study. The parameters we compared and their final (default) values were:

- *minsplit: 20.* The minimum number of observations in a node for which the routine will even try to compute a split.
- *minbucket: minsplit/3.* The minimum number of observations in a terminal node.
- *xval: 10.* The number of cross-validations to be done.
- *cp: 0.01.* The threshold complexity parameter.

The Cox model was created using the R survival package [15]. Each model was then converted into predicting three risk groups by indroducing two cuts on the estimated regression exponent, one for low risk and one for high risk respectively. These cuts were determined as to give exactly the same risk group sizes to those of Rpart (Fig 1).

### ANN models for risk group detection

Classification models, based on ANN, are constructed to predict the risk group categorization of individual patients. Each model is trained to classify either a high-risk or a low-risk group according to a *one-vs-rest* [16] strategy. Combining multiple ANN-models into an ensemble enables classification into a third intermediate risk-group.

The objective function during training is the area under the survival curve for the corresponding risk group. An ANN-model constructed to identify a low (high) risk group will maximize (minimize) the area under the survival curve during training. The area under the survival curve is well-defined and efficient to calculate in 𝓞(*N*) time:

$$\begin{array}{c} A=\sum_{i=0}^{N-1}S_{i}(t_{i+1}-t_{i}) \end{array}$$ (1)

where *S*_*i*_ is the survival rate just before time *t*_*i*_ and *N* is the number of time points. Possible values range between 0 and *t*_*N*_ (the last time point with survival rate 1). Since the maximum is known it is easy to construct *A*′ when minimizing the area:

$$\begin{array}{c} A^{\prime }=t_{N}-A \end{array}$$ (2)

**Fig 2:**
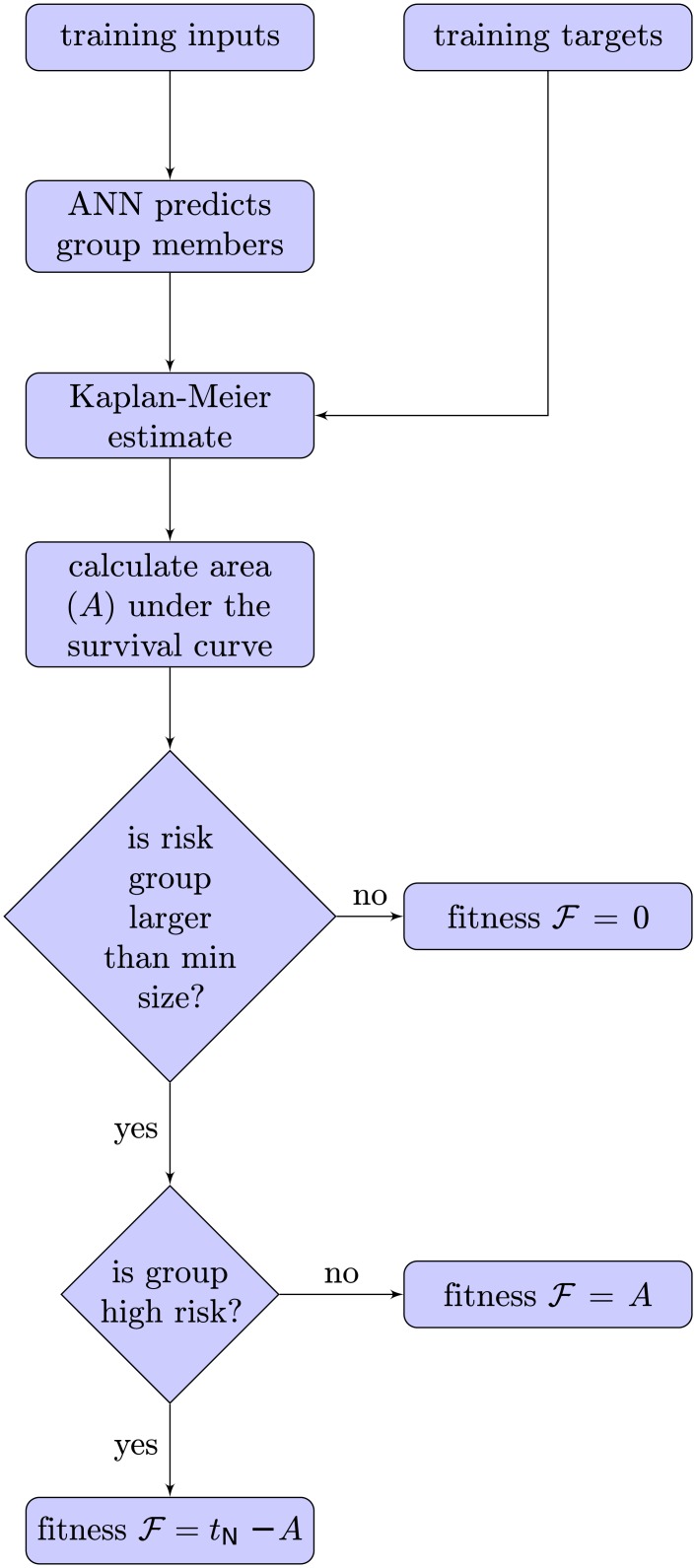
How the fitness of an ANN is calculated during training. The genetic training process strives to optimize the fitness of a population of ANNs. The fitness of each member of the population is determined by calculating the area under the survival curve of its predicted group. If the goal is to identify a low-risk grouping, then the area is maximized. If the goal is to identify a high-risk grouping, then the area is minimized. If the predicted group is not large enough, then a fitness of zero is awarded to the ANN.

There is nothing in this objective function which takes group size into account. This can result in a situation where the optimization algorithm will drive the ANN-model to identify a single high or low-risk individual, and thus coming very close to achieving the maximum or minimum survival area. To avoid this, a hard minimum group size (*m*) constraint was implemented such that a score of zero (higher is better) was given to ANN-models violating this constraint:

$$\begin{array}{c} F=\{\begin{array}{cc} A\;\text{or}\;A^{\prime } & \text{if}\;M\geq m\\ 0 & \text{otherwise} \end{array} \end{array}$$ (3)

where *M* is the number of patients that the ANN model classifies to be part of the risk group. The minimum group size *m* was set to match the size of the groups given by Rpart (see section on Rpart and Fig 1). The difficulty of computing gradients for this objective function requires an alternative to the standard back-propagation learning algorithm for ANNs. A learning procedure based on a genetic algorithm, similar to our previous work on maximizing the concordance index [3], allows the area under the survival curve to be used directly as the objective function during training. The process is illustrated in Fig 2. Details of the genetic algorithm used in this study can be found in the next section.

The individual ANN-models were implemented as multi-layer perceptrons with a single hidden layer (hyperbolic activation functions) and an output layer encoding the risk groups. In our case a binary answer (yes/no) to the question “is this individual part of the group or not?”, the group being either high-risk or low-risk. Note that this is not the same as asking “high or low risk”. The objective of each ANN is only to identify the best high-risk or low-risk group it can, not both. Appropriate training parameters for the genetic algorithm and number of hidden neurons were determined by running repeated 3-fold cross-validation on the training data. We selected values which seemed to work well for all data sets. This set the number of hidden nodes to four, indicating a rather limited complexity of the data sets.

**Fig 3:**
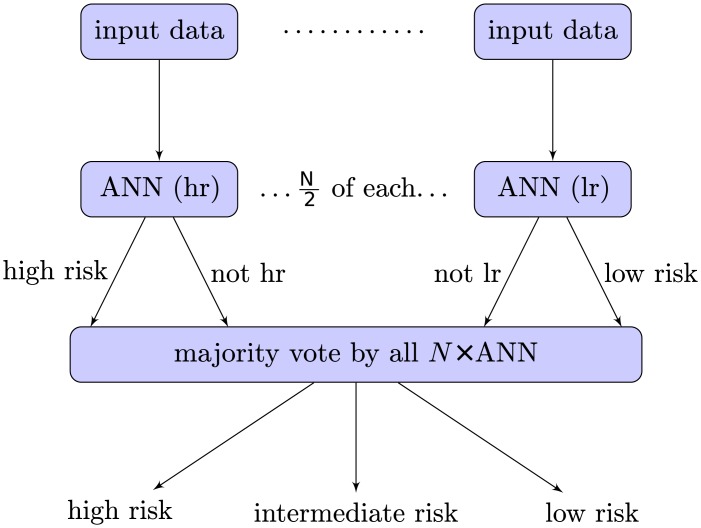
Predicting the grouping for a patient. Each ANN is trained to identify either high risk or low risk individuals. When combined into an ensemble, majority voting determines if the patient is classified as high risk, low risk, or intermediate risk. *N*/2 is chosen to be odd, in order to avoid ties in each subgroup.

Many individual ANN-models were trained and then combined to form an ensemble model, with the ability to answer to “high, low, or intermediate risk group?”. The size of the ensemble was 34 where half of the models were trained to identify a high-risk group and the other half a low-risk group. Each ANN calculates its output: *high* or *not*, *low* or *not*. The final ensemble output is “intermediate” either if the majority vote for both subgroups is *not*, or if there is a tie between *high* and *low* votes. Otherwise, the group with the most votes between *high* and *low* wins. Note that the number of networks is chosen such that each subgroup has an odd number of members to avoid a tie between *not* and anything else. This process is illustrated in Fig 3. Bagging [17] was used to diversify the ensemble members during training.

### Genetic training procedure

A genetic algorithm (sometimes also referred to as an evolutionary algorithm) is an optimization algorithm which simulates *survival of the fittest*. The procedure can be outlined as follows:

1. *Initialize the population.* *N*_*P*_ individual ANNs with random weights are generated. We found that keeping the connection structure constant to be best, but it can be allowed vary. The fitness of each ANN is calculated according to Fig 2.
2. *Select two parents.* Selection is done with the tournament method, where two ANNs are picked uniformly at random and the one with better fitness is selected. The procedure is repeated to select the second parent.
3. *Crossover.* As a first step, the two parents are copied to form two children which are exact clones of the parents. Then with probability *P*_*C*_, two pivot points are randomly selected along the genome vector. The section between the two pivot points is exchanged between the children. With probability 1 − *P*_*C*_, no crossover is performed and the children remain clones for the next step.
4. *Mutation.* For each child, each weight is independently subjected to mutation with probability *P*_*M*_. Mutation of a weight, *ω* = *ω* + *R*, is done by adding a gaussian random number *R* with standard deviation *M*_std_ and zero mean.
5. *Insert into population.* The fitness values of the children are calculated according to Fig 2 and then the children and the parents are re-inserted into the population. To keep the population size *N*_*P*_ constant, the two worst performing ANNs in the population are discarded.
6. Go to Eq 2, unless enough generations *N*_*G*_ have passed. One generation is said to have elapsed when *N*_*P*_ children have been born. If done, then the final result is the single network with the best fitness observed during training.

Each step could be done in multiple ways. Selection can for example be done with roulette selection or with geometric probability. Crossover can be done with only one pivot point, or more, or even completely uniformly at random. Mutation could additionally be done on the connection level between the neurons in the ANNs. Insertion could replace the parents, instead of re-introducing them, or allow the population size to vary. For discussions on the different methods please see [18] and [19].

Many such methods, and their associated parameter values, were compared with repeated cross-validation runs on the training data. Several of the methods had negligible differences so a different method for crossover would likely have performed equally well for example. Each data set produced similar values so we chose to use the same parameters for all data sets. The final parameter values are presented in Table 1.

**Table 1:** Parameters used for the genetic training procedure.

*N*_*P*_	*P*_*C*_	*P*_*M*_	*M*_std_	*N*_*G*_
200	0.75	0.5	1.5	1000

### Data

All data sets were subject to the same pre-processing. First, suitable categorical variables were divided into binary variables (one for each category). In case of missing data, the variable’s mean was used as the imputation value. This seemed sufficient considering we are only interested in the relative performance of the different models. The variables were normalized to have zero mean and unit standard deviation. Finally, 1/4 of each data set was randomly selected (stratified on censoring) and labeled as test data and the rest as training data. All parameter tuning and cross-validation runs were made on the training data. The test data was only used once all models were configured. We compared the models on five data sets, all of which are publicly available and described in more detail in the *survival* package [15] in R.

#### colon

One of the first successful trials of adjuvant chemotherapy for colon cancer [20]. Consists of 929 patients, 461 (50%) of which were censored before recurrence. The target variable is *days until recurrence* and the 11 input features are: type of treatment, sex, age, obstruction of colon by tumor, perforation of colon, adherence to nearby organs, number of lymph nodes with detectable cancer, differentiation of tumor, extent of local spread, time from surgery to registration (short/long), and more than 4 positive lymph nodes (yes/no).

#### flchain

A study of the relationship between serum free light chain and mortality [21]. In total 7871 patients are included with 5705 (72%) patients censored (still alive at last contact date). The target variable is *days until death* and the 7 input features are: age, sex, kappa portion, lambda portion, FLC group, serum creatine, and if diagnosed with monoclonal gammapothy.

#### nwtco

From the National Wilm’s Tumor Study [22]. 4028 patients where 3457 (86%) are censored before relapse. The target variable is *days to relapse* and it contains 4 input features: histology from local institution, histology from central lab, age, and disease stage.

#### pbc

A randomized trial in primary biliary cirrhosis (PBC) of the liver at the Mayo Clinic [23]. The randomized trial consisted of 312 patients where 187 (60%) were censored. The target variable is *days until death* and the 17 input features are: type of treatment, age, sex, presence of ascites, presence of hepatomegaly or enlarged liver, blood vessel malformations in the skin, presence of edema, serum bilirunbin, serum cholesterol, serum albumin, urine copper, alkaline phosphotase, aspartate aminotransferase, triglycerides, platelet count, blood clotting time, and histologic stage of disease.

#### lung

Originates from the North Central Cancer Treatment Group [24] and consists of 228 patients with advanced lung cancer where 63 patients (28%) were censored. The target variable is *survival time in days* and the 7 input features are: age, sex, ECOG performance score, Karnofsky performance score by physician, Karnofsky performance score by patient, calories consumed at meals, and weight loss in the last six months.

## Results

After initial determination of model parameters (on the training data), all models were trained on the entire training set and tested on the test data. Furthermore, using the same model parameters, 3-fold cross-validation repeated 10 times was performed on the training data to compare the consistency of the models. Note that the validation sets are of equal size to the test sets (1/3 of training set = 1/4 of total data). To analyze the high multiplicity of survival curves produced in cross-validation, we chose to focus on some key properties of the survival curves and present the validation results as box and whisker plots. Low and high-risk groups are all presented separately for each model. Boxes indicate the range between the first and third quartiles, and the line inside marks the second quartile (median). Whiskers then extend to at most 1.5 × (*Q*3 − *Q*1) and any data points beyond that are considered outliers and marked as dots. For the test sets however, the results are presented as survival curves. The cross-validation results are presented first.

**Fig 4:**
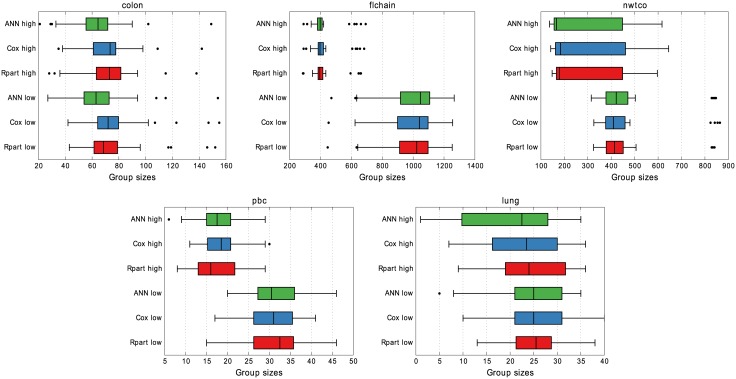
Group sizes in 10 × 3 cross-validation. While the median group size is very similar for all models, ANN identifies high-risk groups that are smaller than the other models’ for *lung*, and also for *colon* with low-risk groups.

Fig 4 shows the group sizes on the validation sets. Both Cox and ANN are configured to produce the same group sizes on the training data as Rpart and this is quite consistently carried over to the validation sets. The medians, boxes, and even outliers are all quite similar thus enabling a comparison of the other properties in a meaningful way.

**Fig 5:**
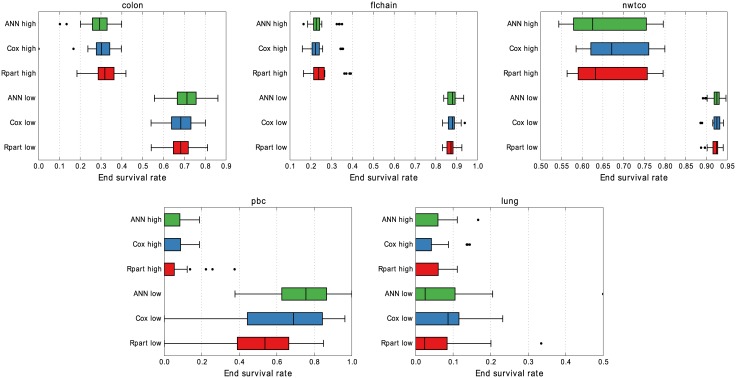
End survival rate in 10 × 3 cross-validation. The distribution of survival rate for the high-risk groups are very similar for all three models, with median values of zero on *pbc* and *lung*. On *pbc*, our ANN approach displays consistently better results for the low-risk group compared to both Cox and Rpart.

**Fig 6:**
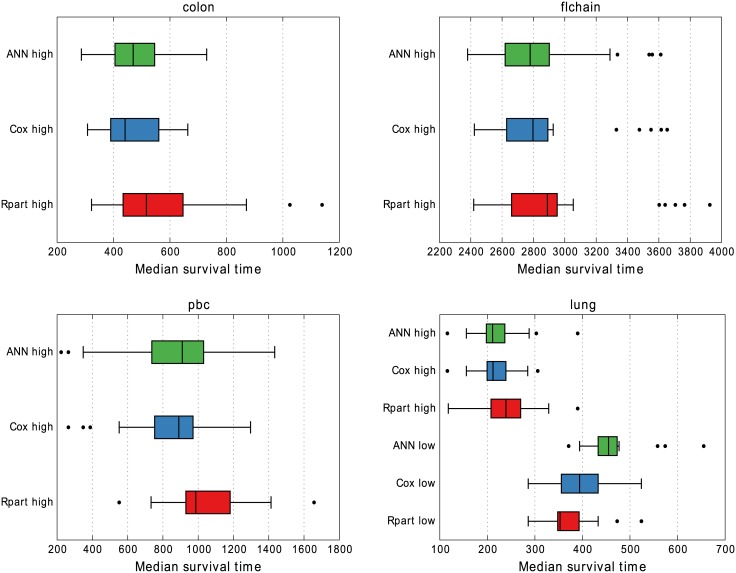
Median survival time in 10 × 3 cross-validation. This is only possible to compute if a group’s survival rate reaches 0.5, which no grouping in *nwtco* did. Groupings in *lung* had so poor survival that even the low-risk groups could be included.

Results do not differ much in Fig 5 either where the end survival rate is compared. One difference is that our ANN approach is consistently better at predicting low-risk groups on *pbc* in terms of end survival rate. Both Cox and Rpart have lower medians and lower range extends to zero whereas ANN only extends to about 0.4. For the same data set, Rpart produces a slightly tighter range on the high-risk groups. The median survival times for the applicable groups are presented in Fig 6. For the *nwtco* data the high-risk group does not go below 0.5 in survival, as an effect of many censored events, and is therefore excluded. The *lung* data on the other hand has very poor survival resulting in all low-risk groups approaching zero in survival rate. For low-risk groups, higher is better, and lower is better for the high-risk groups. Both Cox and ANN have median values consistently on the “better” side of Rpart.

**Fig 7:**
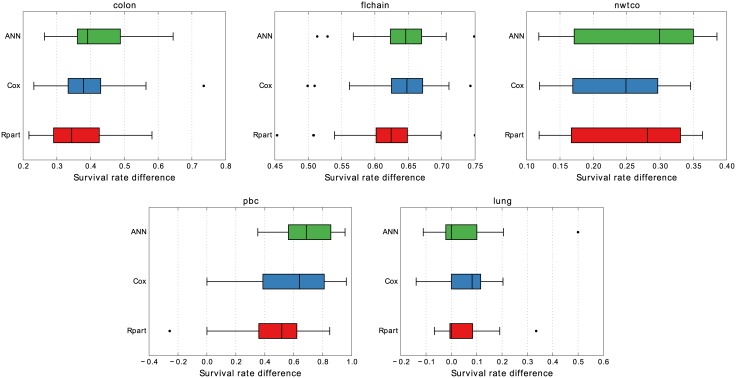
Difference in end survival rate for low and high-risk groups in 10 × 3 cross-validation. If the difference in end survival rate between low and high-risk groups is negative, this means that the survival curve for the low-risk group is below the high-risk group’s curve at the final time point.

To analyze the distance between the high and low-risk survival curves the difference between end survival rate was computed and is presented in Fig 7. A negative value indicates that the curves have crossed and that the low-risk curve is no longer above the high-risk curve. On *pbc*, Rpart displays a single outlier where such a crossing occurred. For the *lung* data however, all the models see a fair amount of crossings. This is not all that surprising given that *lung* has such an extremely poor overall survival rate. One would expect most curves to simply meet at the zero mark, as can be seen in the boxplots (median marks are close to zero). To further analyze the *lung* data, the difference in median survival time between low and high-risk groups was computed for the validation data. Here the ANN model showed the best separation and Rpart the worst.

**Fig 8:**
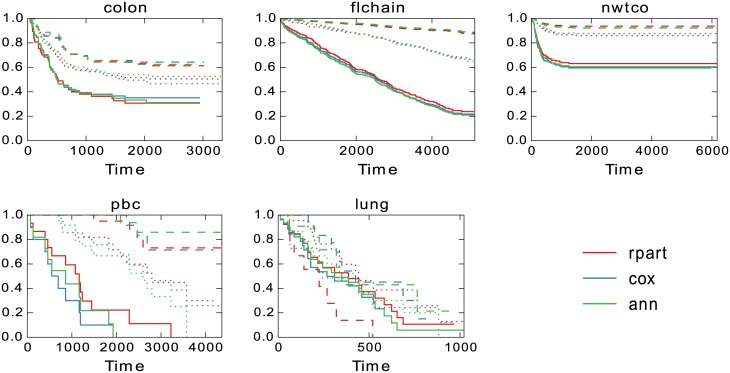
Predictions for the test set. Overall the results for the three models are quite similar. One notable exception is for *lung* where the low risk group predicted by Rpart actually has the worst survival.

The test set results can be found in Fig 8, where survival curves are presented for each model and for each of the determined risk groups. The group sizes are listed in Table 2 and it is apparent that the validation results with very similar sizes also holds for the test set. On *lung* we can see a very early crossing of the curves by Rpart, and the “low-risk” group actually has clearly worse survival than the “high-risk” group. In other cases, the differences are quite small between the predicted groups. A small difference can be found for the low-risk group on *pbc* where the ANN model finds higher end survival together with a slightly larger group size.

**Table 2:** Group sizes for the test set.

Data set	ANN high	Cox high	Rpart high	ANN low	Cox low	Rpart low
*colon*	71	73	57	63	77	70
*flchain*	402	411	415	1091	1078	1101
*nwtco*	163	157	178	451	459	444
*pbc*	11	10	15	27	19	23
*lung*	31	28	26	11	18	9

## Discussion

We have constructed ANN models, based on an ensemble approach, which produce risk groupings of patients. We defined low, high, and intermediate risk groups as that tends to be the clinical practice. Using a genetic algorithm, we have been able to train the ANN models by maximizing (or minimizing) the area under the survival curves. An initial approach instead had the ANN models maximizing the log-rank separation between groups, similar to how Rpart does its group splitting. Our assumption was that the log-rank measure would be higher for larger group sizes (more significant) but this was not the case. Even in an idealized setting, where the groups were constructed by manually selecting the best (or worst) individuals, there was a strong bias towards small group sizes (10–50 for all data sets). We also found that the resulting survival curves were not necessarily well separated in terms of median survival time, or end survival rate. For that reason, we instead decided to optimize the area under the survival curve which implicitly optimizes properties such as median survival time and end survival rate for the risk group at hand.

To control group size and to facilitate comparisons with other models (Cox and Rpart) a lower group size limit was added in the genetic optimization procedure. Cox and ANN models were configured to have the same group sizes on the training data as Rpart since it is the least flexible in terms of group size. The splitting algorithm in Rpart will typically generate some very small groups and employs a minimum split size (20 by default) to compensate just as our ANN approach does. Many of the final groups generated by Rpart are however not statistically different from each other and can thus be combined [13]. To further reduce the number of groups, and enlarge the high/low-risk groups, we manually combined the outer groups until they came closer to a quartile in size. This was performed on a per data set basis because some combinations could not be motivated due to size constraints or statistical differences. For example, the low-risk group generated on *nwtco* was 55% of the entire training data without any merging. But this is more a problem with the data being skewed: *nwtco* has 86% censoring so a large low-risk group was to be expected simply by looking at the data distribution. If medical practice calls for particular group sizes, the flexibility offered by our ANN-based approach when it comes to specifying the expected group size can be an advantage.

The resulting group sizes on validation and test data are presented in Fig 4, and Table 2 respectively. Labeling 1/4 of the data as test, and then doing 3-fold cross-validation on the rest means the test set and validation sets have the same size. The configured training group sizes carry over quite consistently to both the validation and test sets for all the models, a prerequisite for comparing properties of the risk groups.

One such property is the end survival rate for the groups. The predicted risk groups are overall very similar in this regard but two things do stand out. First, on *pbc* in Fig 5, the end survival rate for low-risk groups predicted by the ANN models are always greater than zero while both Cox and Rpart at some point predict zero survival for the low-risk group. Second, on *lung* the predictions by Rpart on the test data in Fig 8 are completely off. The low-risk reaches zero survival about half-way through while the high-risk group has a non-zero survival. All models have difficulty with this data set as can be seen by the very close survival curves for all the groups.

The performance by Rpart on *lung* is an example of crossing survival curves, which indicates miss-classification. The curves cross very early in Fig 8 for Rpart but a closer inspection reveals that all models exhibit crossing for some group towards the later survival times. This is probably largely due to the very small size of the *lung* data set. As seen in Table 2 the low and high-risk groups range from 9 to 31 in size. Still, we chose to investigate how common crossing survival curves were for the data sets. Fig 7 shows that while Rpart did have a single crossing event on *pbc*, it generally only happens on *lung* and it does so for all models. Not entirely surprising given the extremely small size of the data set. A closer look reveals that ANN has the best separation of the curves in terms of median survival time and Cox slightly better than Rpart.

For median survival times in general, the results in Fig 6 are quite similar. Rpart has slightly wider distribution of results on *colon* but ANN has wider distribution on *flchain*. For the smaller data sets, Rpart’s high-risk group has higher (worse) values on *pbc* and the ANNs’ low-risk group has higher (better) values for *lung*. These data sets are small but the results are consistent between cross-validation and test-set, indicating that Rpart is less robust for smaller data sets.

Some might argue that a point in favor of Rpart is the interpretability of the decision tree. This is certainly true for very small data sets where the decision tree only has a depth of two or three (and a similar amount of leaf-nodes) but holds little merit on larger data sets where the decision tree by necessity is deeper. As illustrated by Banerjee et al. [13], several of the leaf-nodes will not be significantly different which means that there are multiple paths to the same output. At this point, Rpart is just as interpretable as an ANN, or in fact any non-linear model. Non-linear models are difficult to interpret regardless of their representation.

Another limitation of the proposed method can be found when predicting more than three risk groups. The current approach of a binary classification (high/low risk versus not high/low risk) together with an ensemble approach is not suitable for more than three risk groups. Furthermore, the handling of missing values for the different medical data sets in the experiments was the simplest possible. More advanced imputation techniques could have improved the overall ability of all methods to predict good risk groups.

The overall result on the test sets show comparable performance for all three models, although one can identify small differences and outlier results for some models. In our opinion the ANN approach offers an advantage of being non-linear and flexible in the choice of group size. It is worth noting that none of the data sets used here showed strong non-linear effects since the performance of the Cox models were comparable with both Rpart and ANN. Another possible merit of ANN not investigated in this study, is that the distribution of votes in the ensemble could be a cheap estimate of confidence intervals for individual risk group classification.
